# Internal Uterine Hemorrhage

**Published:** 1840

**Authors:** 


### INTERNAL UTERINE HEMORRHAGE.

*Dr. Dunglison* copies the following excellent lecture, by J. T. Ingleby, M. D. on Internal Uterine Hemorrhage, from the London Lancet for January, 1840.

On the present occasion I wish to call your attention to the subject of uterine hemorrhage, in one of its most peculiar and imminently dangerous forms—I mean hemorrhage accompanied by a detachment of the placenta, together with an infiltration of blood in its substance, constituting what has been termed *placentary apoplexy*, and arising about the close of pregnancy. It may occur either independently of labor, or whilst labor is progressing. The one object I have in view being to have the matter fully understood by you all, I will enter upon the subject without further preface, studying only plainness and clearness of description.

I have said I wish to call your attention to hemorrhage, accompanied with detachment of the placenta. First of all, then, I would have you observe, that the detachment usually commences *about the centre* of the mass, and extends to every part of it, the edge excepted, which maintains its natural apposition; consequently, a large quantity of blood soon becomes confined between the placental and uterine surfaces. The uterine tumour at these points becomes raised in proportion to the amount of effusion, its rapid augmentation constituting the most striking feature in the case. But it is important to observe that the effusion may commence *any where between the centre of the placenta and its edge*, which almost necessarily becomes more or less detached, so that whilst a large coagulum is confined, partly underneath the placenta and partly exterior to the membranes, the liquid blood continues detaching the membranes until it reaches the vagina: I have seen both forms of hemorrhage. The first, or *concealed* form, from its greater liability to deceive the practitioner than the second, may be regarded as the most dangerous case, although the extent of hemorrhage *in this form* is less considerable than *in the other*. I shall presently state a remarkable exception; but this, as a rule, is generally correct.

Case.— Mrs. B. has ten or eleven children, and was subjected to my professional notice when ahout nine months advanced in pregnancy. During the night of Friday, Sept. 8, a discharge of blood, both clotted and fluid, occurred several times; and at three oclock, on Saturday morning, my friend Mr. Rice was called to see her. Although she had reached the full period of pregnancy, no pain took place until subsequently to the attack of hemorrhage, and the degree of pain which then arose was inconsiderable. The amount of hemorrhage was trifling, three napkins only having been stained, but the depression of the general system had progressively increased, and was at that time most alarming. On examining the uterine tumour, Mr. Rice's attention was immediately directed to a very marked singularity in its shape, the shape being exceedingly pointed, having its long diameter in the antero-posterior direction. I accompanied Mr. Rice to the patient at eight o'clock A. M. There was great prostration of strength, an exsanguine countenance, and the gaping which attends the state of syncope. The pulse was feeble and slow, and the defined eminence which occupied the summit of the uterine tumour, and for some extent around it, was much more elastic than the surrounding parts. Under a strong conviction—a conviction previously entertained by Mr. Rice—that the symptoms depended upon a large internal effusion of blood, I recommended immediate delivery; and, as Mr. Rice entertained similar views of the case, he undertook the operation without delay. Although there had been no regular labor-pain, the uterine orifice was moderately well dilated, and the membranes were sufficiently distended to admit of the bag being very easily ruptured. The circumstance of the membranes being *distended*, deserves your notice in reference to the manner in which the liquor amnii acquired its bloody appearance. On the membranes being ruptured, a large amount of deep colored, bloody fluid instantly rushed out of the vagina. During the delivery of the lower extremities, a quantity of tolerably consistent blood, mixed with small clots, continued to escape, and, on the completion of the delivery, an immense clot was expelled somewhat forcibly. This was rapidly followed by the placenta having, upon its uterine surface, and within about a third of its texture, a mass of coagulated blood. The coagula were so interwoven with the parts as to admit only of very partial removal, and this not without tearing the placenta. The shape of the placenta was sacculated at such of its parts as was not infiltrated, but merely covered by clot, the greater part of the blood having been confined in the sac. Brandy and the tincture of ergot, in combination, were resorted to several times during the delivery with excellent effect in sustaining the pulse, and securing an efficient uterine contraction. The patient would necessarily have been greatly alarmed by the vast disgorgement of blood from the uterus, had we not prepared her mind for the occurrence. As there was no direct escape of blood from the general system, there was no actual shock; rather, indeed, a revival from impending death to a state of comparative security. The large clot, of which I spoke above, weighed two pounds; and the liquid blood, such as, at least, could be collected, weighed two pounds more. Making allowance then, for the blood which had become mixed with the liquor amnii, as well as for the blood which had escaped on the bed and napkins during the night, the actual loss, within six hours, must have been *upwards of five pounds at least.* It is certain that the uterus contained, at the moment of delivery, upwards of four pounds. I need scarcely say, that under so large and so sudden an effusion the fœtal circulation would very soon cease.

And now, gentlemen, let us inquire what practical inferences can be deduced from this narrative? Let us examine it in several points of view.

1st. *The Mode of Attack.*—The attack occurred *suddenly*, and was not the result of external injury—a very probable *means* of producing not only separation, but laceration of the placenta,* and laceration even of the uterus itself. Each of these injuries I have personally witnessed as the result of physical force, but in this case there was no pretence whatever for supposing the existence of such a cause. The circumstances which occasioned the separation of parts, and consequently the effusion of blood, can however only be conjectured. We can only say, with any certainty, that the effusion must have proceeded from a very large vessel.

2dly. *The Symptoms.*—The symptoms were both *local* and *constitutional.* The former comprising the hemorrhage, which appeared external to the body—the shape of the uterine tumour—the *sensation* imparted to the hand when placed over its most projecting part, (a sensation of undue elasticity when compared with the very slight elasticity which characterized the other parts of the uterine tumour,) and the peculiar character of the pains, the feeling being of distress from distention, rather than of suffering from contraction. Hence it is impossible to resist the conclusion, that the pains arose as a consequence of the effusion. It has been already observed, that the pains were preceded by visible hemorrhage. The

---

* The case related by Mr. Wildsmith is a striking instance of this kind. The patient died during pregnancy, and on examination, P. M., a clot of blood was discovered, weighing eighteen ounces, at the anterior part of the fundus of the womb, and the placenta was lacerated.—*See North of England Med. and Surg. Jour.* vol. i. p. 446.

*constitutional symptoms* were merely those that are common to all severe hemorrhages, viz: torpor, drowsiness, repeated syncope, a pallid countenance, a feeble slow pulse, gaping, and coldness of skin.

I have only one remark to offer, in reference to the depression of the system, viz; that it was very great, and yet altogether disproportionate to the amount of visible discharge. Still the fact of an existing visible hemorrhage would naturally impress the mind with the conviction, that the sinking of the vital powers and the hemorrhage, slight as it was, must have had an important connection. In this respect, the evidence, if not altogether conclusive in the instance before us, was far more conclusive than characterized several fatal cases of a similar kind.

3dly. *The condition of the membranes and the liquor amnii,* is a point not altogether destitute of practical interest. There was nothing peculiar in the state of the os uteri; it was relaxed and partially open, but these characters are common to the uterine orifice at the close of pregnancy, in a person having had several children, as was Mrs. B.'s case. The membranes were apparently entire, the presenting portion being moderately distended with fluid. *The liquor amnii* had a very bloody appearance, and gushed out very forcibly on the bag being ruptured. In its passage through the vagina, it is indeed usual for the liquor amnii to acquire a stain from the blood which may be lodging there, but here the fluid was uniformly bloody, the color being almost as deep as the blood itself. The precise cause of this is not easily explained. The fœtal side of the placenta was perfect, consequently the stain must have taken place, either from a slight tear at the edge of the placenta, (a circumstance which would not prevent the presenting part of the sac from being moderately distended,) or it must have been the result of transudation. I incline to the former opinion, the *period* of transudation having been very short, although the transuding *surface*, from the size of the coagulum, was considerable. Certainly the fact of the liquor amnii not containing coagula may be supposed rather to favor the view last suggested. One is naturally led, therefore, to make an inquiry as to the source of the blood. Did it proceed from the placenta itself, or from the vessels of the uterus in connection with it? What are the probabilities? The placenta was very pulpy throughout, and about one third of the mass, from the edge towards the centre, was so completely infiltrated with blood, as to render the removal of the clots impracticable without breaking up the structure of the placenta itself. *Consequently it was impossible to detect any*

*open vessel.* I am disposed to think, that the blood proceeded from the uterine system and not from the placental, and I will give my reasons for this opinion. As already observed, the infiltration was very limited in its extent, although it pervaded the whole thickness of the mass. Now, had the blood emanated from the interior of the placenta it could only have proceeded from a large vessel belonging to the umbilical system, and it is more than probable that the *greater part* of the placental mass would have been infiltrated. Moreover, had the case been so, I think the extravasation would have been apparent through the coverings of the fœtal surface. But it was not apparent in any degree. Neither is it probable that the blood, after traversing the interior of the mass, could have retained its fluidity sufficiently long to have passed in such large quantities into the uterine cavity. I can only account for the infiltration by the supposition of a breach of surface having taken place in the placenta, whilst the extent of detachment was slight.

Such is as complete an outline of this remarkable case as it is possible to set before you in a lecture; and, considering the danger young practitioners are in, of forming a wrong judgment upon the symptoms, and the danger of improper treatment to the patient, I do most earnestly press upon you the duty of a careful study of this and similar cases. I will now lay before you all the information I have been able to obtain on this particular kind of hemorrhage, and a case or two not previously recorded. My own work on "Hemorrhage," contains scarcely anything on the subject; indeed the records respecting it are very scanty. Dr. Simpson's elaborate paper, on "Diseases of the Placenta," contains several references to the class of cases immediately before us; I recommend you to peruse this paper carefully. It evinces great research, and is replete with practical information.* Dr. Merriman alludes very briefly to the circumstance, that syncope, or even death itself, may be occasioned by an effusion of blood between the uterus and placenta, whilst "there may be very little appearance of discharge from the vagina." Dr. Blundell, also, in adverting, in general terms, to instances of death occurring suddenly in the last months of pregnancy, observes: "On laying open the body after death, two or three pounds of blood may be discovered within the cavity of the uterus, and this, too, although there may have been no external bleeding." The first case which I have met with is related by the

* See "Edin. Med. and Chir. Jour." for April 1, 1836.

celebrated Albinus,* where only the central part of the placenta being loosened, a large quantity of coagulated blood was lodged between it and the uterus, as it were, in a bag, and, consequently, not a drop was discharged per vaginam. "Had the nature of the case been understood, (observes Albinus,) the patient might have been saved by rupturing the membranes, and delivering immediately." Four cases are related by M. Baudelocque. The mother was saved in three of the cases, but the child perished in each of them. In one of these the quantity of blood behind the placenta was estimated at four or five *palettes*.† Baudelocque relates a fifth case; the hemorrhage, however, took place within the membranes, and not behind the placenta. Two cases are related by M. De Laforterie; the first case terminated fatally, after twelve hours' labor-pain, and before competent assistance could be obtained.‡ M. De Laforterie, however, performed the Cæsarean operation, and, on opening the fundis uteri, a pound and a half of liquid black blood immediately gushed out, which had been contained in a sac, between the placenta and the uterine surface, the centre of the placenta having been detached, while the edge remained adherent. The child was extracted alive, but speedily died. In the second case, the quantity of blood is said to have measured three French *chopines*.||

Mr. Saumarez adduces a well authenticated, but fatal case, of this form of hemorrhage. There was no discharge per vaginam. On examination, P. M., the placenta was every where detached, excepting its edges, which were "completely adherent, forming a kind of *cul de sac*, into which blood had been poured to the amount of a pint and a half, which had become coagulated within the cavity thus formed." The patient was also attended by Drs. Denman and Denison.§ Dr. Hamilton describes two cases. In the first, premature labor occurred spontaneously. "In the central part of the placenta a strong coagulum of blood, the size of an afternoon teacup, was discovered. The adhesion of the edges of the placenta, had saved the patient." The result of the second case was less fortunate. The symptoms were those of collapse, and

* "Annot. Acad.," lib. i, c. 10., p. 56.

† The *palette* contains four ounces.—Ed. L.

‡ See "Jour, Gen.," tom. 29, p. 384, and quoted in Mons. C. A. Baudelocque's "Trait des Hæmorrhages Internes de l'Uterus."

|| The *chopine* contains about an English pint.—Ed. L.

§ See No. 6, "New Lond. Med. and Phy. Jour.," p. 335.

"the lady felt as if she were going to burst; there was no discharge from the uterus, and no symptoms of labor. Immediate delivery was accomplished, by passing the hand into the uterus, and a dead infant was extracted, which was followed by an immense quantity of coagulated blood and the placenta. The patient almost instantly expired."* I now refer you to a very clear and concise paper on this subject, illustrated by a particularly well marked case, by my friend, Mr. J. M. Coley.† The effusion was characterised by a sudden enlargement of the uterine tumour, together with a sensation of pain, as though the abdomen would burst, and by frightful collapse of the vital powers. There was no discharge whatever from the vagina. Delivery was accomplished by rupturing the membranes, and the administration of ergot; and, on the expulsion of the placenta, it was ascertained that blood had been effused, between the placenta and the uterus, to the amount of two pounds, and also extravasated within the placental cells.

I shall now mention two cases which have presented themselves to my notice. Some weeks ago I was requested to see a woman, reported to be in convulsions. Before I could reach the house she had expired—labor was supposed to have commenced the preceding evening. A respectable surgeon employed in the case, finding the pains excessively feeble, and the system much depressed, ruptured the membranes. The liquor amnii was colorless, and no hemorrhage was visible at any time. The body was examined, P. M., by the surgeon just alluded to, assisted by my friend Mr. Wickenden and myself. The form of the uterine tumour was strikingly conical. On cutting through the uterine parietes, so as barely to receive the end of the scalpel—fluid blood rushed out like the stream in venesection. By means of a sponge 60 ounces of liquid blood were collected, and, on enlarging the aperture, a coagulum was removed which weighed 61 ounces, the whole comprising 121 ounces of blood; the placental edge was still adherent, so that there had been no escape of blood underneath the membrane. The circumference of the placenta was inordinately large. The other case, which, in several respects, is unlike the one just reported, derives an interest from the amount of blood being very trivial, and yet proving fatal to life. A young woman, from three to four months pregnant, having just eaten breakfast, went up stairs in per-

---

* See "Prac. Observ." part ii., p. 235—6.

† See Lancet for 9th January, 1840, p. 498.

fect health and spirits to make her bed. She returned very quickly, complaining of feeling very ill, sat down in a chair, and expired. An inquest was held, and the body reported to be perfectly healthy. On the close of the inquest, the impregnated uterus was brought to me unopened, as a fine specimen of natural pregnancy. On opening it a portion of clot of blood appeared to view. It had lacerated the chorion to a a very slight extent only; but, on removing it from its bed, between the amnion and the chorion (a most singular situation to contain so large an effusion, of which Baudelocque gives no example, but refers to several examples shown him by Professor Deneux.) it was found to weigh four ounces. A slight stain was also observed on the woman's linen, which from its dampness must have been recently produced. She died in a state of syncope. The nervous system must have received a severe shock at the moment of the laceration, for, of itself, so small an amount of blood could scarcely bring life into danger, even in the sitting posture. The sudden uterine distention might have had an important connection with the fatal depression of the action of the heart. The indications of treatment, in cases attended with a large internal effusion of blood, are very simple, viz: evacuating the uterus and securing its effective contractions. In the form of hemorrhage, termed "accidental," the mere rupture of the membranes is the practice generally pursued, and with marked success—the hemorrhage ceases, and labor presently comes on; but, in an exigent case of internal hemorrhage, the same reliance cannot be placed on this simple operation. The objections are threefold.

1st. The chance of the uterine contractions, either not coming on, or proving inadequate to constringe the bleeding vessels—a highly probable supposition, considering the mass of blood which may intervene between the uterus and the membranes.

2dly. The uncertainty of the period of time which elapses previously to contractions arising.

3dly. The impossibility of determining at the moment, whether or not the hemorrhage is arrested, our opinion being regulated entirely by the constitutional symptoms.

Mr. Coley's patient was treated by the rupture of the membranes merely, and the administration of the ergot of rye; pains came on in three hours and fifty minutes afterwards, and the child was expelled by the natural powers. Mr. Coley was deterred from turning the child by "the death-like state of collapse."

In a case already described, attended with an effusion of 121 ounces of blood, the rupture of the membranes had no effect whatever in producing uterine action, I do not recollect whether or not the ergot was given. Hasty conclusions, derived from solitary cases, are often incorrect, and I would not be understood to say that a case may not occur like Mr. Coley's—the patient being almost *in articulo mortis*—where the milder practice might not be preferable to the sudden evacuation of the womb. Indeed, as I have already stated, Dr. Hamilton's patient died immediately upon artificial delivery. Still, whenever there is reason to believe the hemorrhage is going on, the evacuation of the uterus, by turning the child, should be undertaken at any risk, for it is very probable, that during the time we are waiting for the natural action of the womb, an additional quantity of blood may be gradually pouring out, calculated to terminate life. If this be true, the plug must indeed be a dangerous remedy in such cases, and yet Mr. Baudelocque recommends it as a temporary measure, provided the os uteri is too rigid to admit of the hand. Nevertheless, he enforces the practice of immediate delivery as early as possible, and happily the os uteri will almost always be found abundantly relaxed for the purpose. Sufficient evidence has been adduced to show you the great danger of all cases like the present, and the inevitable consequences of indecision. Had Mr. Rice been a less thoughtful and cautious practitioner, than he is known to be, his patient would most certainly have perished—for like cases of placental presentation, nature is unequal to the emergency, and art has the preeminence. Amidst much that arises to discourage us in the exercise of this most responsible department of medicine, we now and then possess the certain conviction of having been, under Providence, directly instrumental in the preservation of human life—perhaps (as in this case) preserving the life of the mother of many children.

---

*On the causes of Sudden Death.—By Alphonse Devergie.** It is the common opinion that apoplexy is the most frequent cause of sudden death. M. A. Devergie has endeavored to ascertain how far this opinion is founded on truth, and has found that sudden death from affection of the brain is rare. Of forty cases which he has examined he has met with four

---

* Annales d'Hygiene Publique; July, 1838, and Edinb. Med. and Surg. Jour., January, 1840, p. 242.

only in which death resulted from an affection of the brain ; three in which there was congestion of the brain and spinal marrow ; and twelve in which the brain and lungs were simultaneously affected. Sudden death from an affection of the lungs alone is the most common. M. Devergie met with twelve cases of this out of forty ; and if to these we add the twelve examples of sudden death in which the lungs and brain were both affected, we shall have twenty-four out of forty in which the lungs were affected in cases of sudden death. Death from affections of the heart was the most rare. M. Devergie met with it only three times.

It results from these researches that, if arranged according to the order of their frequency, sudden deaths are occasioned, 1. from affections of the lungs ; 2. of the lungs and brain ; 3. of the brain and spinal marrow ; 4. from hemorrhage ; 5. from an affection of the heart. It is consequently an error to regard apoplexy, that is circumscribed cerebral hemorrhage, as the most common cause of sudden death, since in the forty cases M. Devergie observed an apoplectic effusion of blood only once. Sanguineous congestions of the meninges should not be ranked among cerebral hemorrhages. M. Devergie further ascertained that sudden deaths were more frequent during winter, and more common in men than women. Among the forty deaths noted only five were of women. He also observed that sudden deaths occur chiefly among persons from forty to fifty, and sixty to seventy years of age.

*Amer. Med. Int., May* 1, 1840.

